# Safety and Feasibility of Transcatheter Edge-to-Edge Repair of Mitral Regurgitation in Cardiac Amyloidosis

**DOI:** 10.1016/j.jacadv.2025.101998

**Published:** 2025-07-17

**Authors:** Julia Vogel, Peter Luedike, Katharina Hellhammer, Stephan Settelmeier, Sophia Jura, Florian Buehning, Tobias Lerchner, Thomas Mondritzki, Alexander Carpinteiro, H. Christian Reinhardt, Florian Schindhelm, Amir Abbas Mahabadi, Tienush Rassaf, Lars Michel

**Affiliations:** a Department of Cardiology and Vascular Medicine, West German Heart and Vascular Center, University Hospital Essen, Essen, Germany; b West German Amyloidosis Center, University Hospital Essen, Essen, Germany; c Department of Cardiology and Intensive Care Medicine, Niels-Stensen-Kliniken, Marienhospital Osnabrück, Osnabrück, Germany; d Department of Health, University of Witten/Herdecke, Witten, Germany; e Department of Hematology and Stem Cell Transplantation, West German Cancer Center, University Hospital Essen, Essen, Germany; f DKTK-German Cancer Consortium, Site Essen-Düsseldorf, Essen, Germany

**Keywords:** cardiac amyloidosis, cardiomyopathy, edge-to-edge-repair, heart failure, HFpEF, mitral regurgitation

## Abstract

**Background:**

Cardiac amyloidosis (CA) is characterized by amyloid deposits in the heart leading to various manifestations including heart failure (HF). CA patients often present with severe mitral regurgitation (MR), complicating management in patients. Transcatheter edge-to-edge repair (TEER) offers an interventional option for patients at high surgical risk.

**Objectives:**

The objective of this study was to assess the safety, efficacy, and clinical outcomes of TEER in patients with CA and severe MR.

**Methods:**

This retrospective study included 27 patients with CA and 81 matched patients with HF without CA and severe MR who underwent TEER. Outcome parameters included technical success, echocardiographic response, laboratory biomarkers, and clinical symptom burden. Follow-up was performed at a median of 103 days (90-144) and included clinical assessment, echocardiography, and laboratory analysis.

**Results:**

Patients had a median age of 79 (75-83) years with 86.1% male and NYHA functional class ≥III in 85.1% prior to intervention. TEER was successful in 100% in both groups. Symptom burden improved in both groups (NYHA functional class I/II in 50.5% vs 57.8%, CA vs HF). MR was reduced, achieving MR ≤2+ in 100%, with a reduction in regurgitant volume in both groups (*P* < 0.001). No procedure-related major adverse events were reported with a 100% 30-day survival rate. Scoring for MR anatomical complexity did not show a difference.

**Conclusions:**

The study demonstrated safety and feasibility of TEER in patients with CA and severe MR, with satisfactory procedure-related outcome and absence of severe adverse events, thus highlighting the potential benefits in CA patients.

Cardiac amyloidosis (CA) is a progressive infiltrative myocardial disease characterized by cardiac amyloid deposits leading to restrictive cardiomyopathy.^1^ Cardiovascular comorbidities include progressive heart failure (HF), arrhythmia, and valvular heart disease.^2^ The incidence of CA is significantly underestimated, with an estimated incidence of 5% to 17% in elderly patients with HF with preserved ejection fraction or aortic valve stenosis.^3^^,^^4^ There are currently over 30 known proteins that can lead to CA and the most common forms are transthyretin (ATTR-CA) and immunoglobulin light chain (AL-CA) amyloidosis.^5^^,^^6^

Guideline-directed medical therapy for HF is often not well tolerated in CA patients due to hypotension, chronotropic incompetence, and autonomous dysfunction.^7^ Specific therapeutics for the treatment of ATTR-CA, such as TTR stabilizers, have already been approved and various new therapeutics are currently in the pipeline.^7^^,^^8^ The treatment of AL amyloidosis is commonly based on cytoreductive strategies for plasma cell dyscrasia.^9^^,^^10^

As CA is frequently associated with significant cardiovascular comorbidities, addressing these conditions is a cornerstone of effective CA therapy. Mitral regurgitation (MR) is common in CA due to atrial enlargement from advanced restriction and diastolic dysfunction and may be further complicated by amyloid deposition affecting the valve itself.^11^ Severe MR significantly impacts morbidity and mortality in CA patients by further decreasing the effective stroke volume (SV).^11^ A conventional surgical approach for MR is often challenged by age, severity of disease, and comorbidities, thus posing a very high risk for the vulnerable collective (Central Illustration).^12^ Therefore, an interventional approach may be the favorable option if technically suitable. Transcatheter edge-to-edge repair (TEER) has emerged as an established alternative to surgical mitral valve (MV) repair in patients with high surgical risk,^13^^,^^14^ but its role in patients with CA remains controversial.^15^ Previous studies have suggested that the presence of CA in patients with MR undergoing TEER is associated with poor outcomes and an increased risk of procedure-related complications due to the altered valve morphology caused by amyloid deposits, more recent data^16^ indicate that procedural success and periprocedural complication rates are similar between patients with CA and those with isolated MR. However, a first prospective study has shown promising data of TEER in complex anatomy.^17^ The withholding of TEER from patients diagnosed with CA and severe MR has the potential to deprive these individuals of an effective treatment option, particularly in light of the limited availability of alternative therapeutic modalities.

**Central Illustration:**
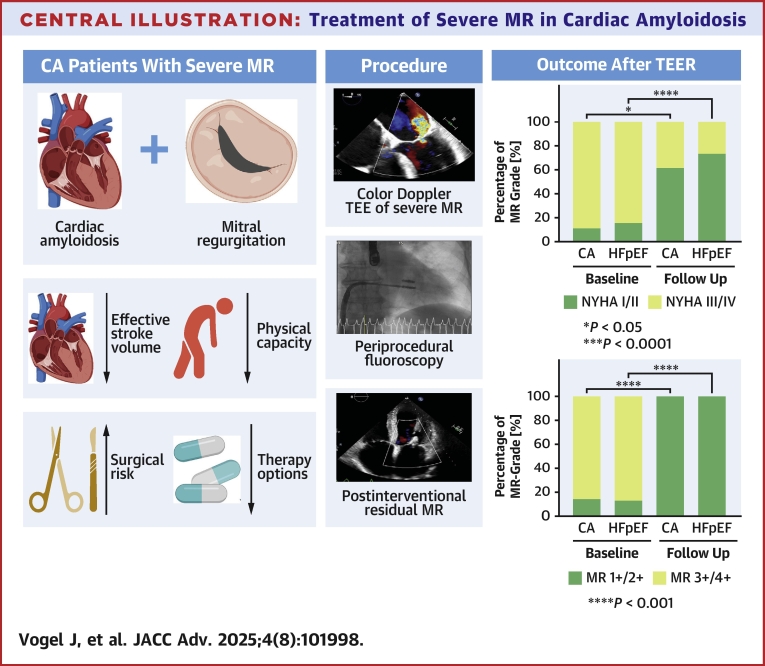
Treatment of Severe MR in Cardiac Amyloidosis (Left) Challenges in CA with MR. (Middle) TEER procedure. (Right) Outcome after TEER. CA = cardiac amyloidosis; HF = heart failure; MR = mitral regurgitation; TEER = transcatheter edge-to-edge repair; TEE = transesophageal echocardiography; HFpEF = HF with preserved ejection fraction.

The aim of this study is to assess the impact of CA on safety, feasibility, and outcome in patients receiving TEER for severe MR.

## Methods

### Study design and population

In this retrospective study, we aimed to investigate the outcome after TEER of severe MR using the PASCAL transcatheter valve repair system (Edwards Lifesciences). All consecutive patients with CA that received TEER for MR from January 2019 to August 2024 at the Department of Cardiology and Vascular Medicine, University Hospital Essen, Germany were included. CA diagnosis was confirmed previously by invasive (cardiac or extracardiac biopsy) and noninvasive (scintigraphy, immunofixation, and/or echocardiography) criteria following current recommendations.^2^^,^^18^ Severe MR was defined by the Guidelines of the American College of Cardiology and American Heart Association.^19^ Anatomical complexity criteria were assessed according to Hausleiter et al.^17^ Matched controls with HF and TEER for MR were included in a 3:1 fashion. Matching was based on age, sex, and left ventricular ejection fraction to ensure comparability between the 2 groups and minimize bias.

Standardized mean differences (SMDs) were calculated to assess baseline balance between CA and control groups beyond the matching variables. We included 27 patients with CA and severe MR and 81 matched control patients with HF without CA. The primary outcome parameters included safety (procedural failure or surgical conversion, bleeding, others), efficacy (technical success), and clinical outcomes (symptom burden and echocardiographic parameters). Echocardiographic data, laboratory values, and clinical outcomes after TEER of the MV in CA patients to those of matched controls. Procedure-related data were extracted from procedural protocols. Survival was assessed based on the last patient contact or death as per medical records. All procedures were performed between March 2019 and August 2024. This study was approved by the ethics committee of the University Duisburg-Essen (23-11500-BO).

### Echocardiography and TEER procedure

All patients underwent routine transthoracic echocardiography (TTE) according to local protocols and international echocardiographic guidelines.^20^ TEER procedures were performed under general anesthesia or conscious sedation with transesophageal echocardiographic and fluoroscopic guidance using the PASCAL, PASCAL Ace, or PASCAL 10 system (Edwards Lifesciences). The devices were introduced through the femoral vein and advanced to the MV by crossing the interatrial septum. All patients were continuously monitored for ≥24 hours post-treatment and underwent echocardiography and sonography of access vessels before discharge.

### Follow-up and statistical analysis

The follow-up period was defined as the median time from the TEER procedure to the first patient contact after procedure, with a median follow-up duration of 103 (90-144) days. A 2-sided *P* value of <0.05 was defined as statistically significant. Kolmogorov-Smirnov statistics were used to assess normal distribution. For normally distributed continuous variables, Student's *t*-test or 1-way analysis of variance for more than 2 groups were applied. For non-normally distributed variables, the Mann-Whitney *U* test or the Kruskal-Wallis test for more than 2 groups were used. Normally distributed variables were documented as mean ± SD. Non-normally distributed variables were documented as median (IQR). Binary variables were compared using the chi-square test. Survival was assessed by Kaplan-Meier analysis and log-rank test. Graphpad Prism 10 (Graphpad Software) and Excel (Microsoft) were used for data analysis.

## Results

### Baseline characteristics

In the CA group, 23 patients with ATTR amyloidosis (wild-type ATTR, n = 22; variant ATTR, n = 1) and 4 patients with AL amyloidosis were included. Staging was performed by National Amyloidosis Centre staging system for ATTR-CA^21^ and Mayo-Clinic staging system for AL-CA^22^^,^^23^ (Table 1).

**Table 1:** Baseline Characteristics and TEER Procedure-Related Data

	All (N = 108)	HF + TEER (n = 81)	CA + TEER (n = 27)	*P* Value
Age (y)	79 (75-83)	79 (75-83)	79 (74-82)	0.81
BMI (kg/m^2^)	25.4 ± 3	25.5 ± 3	25.1 ± 4	0.59
EuroScore II (%)	6.9 ± 6.5	7.0 ± 6.9	6.4 ± 5.2	0.69
Male, n (%)	93 (86.1)	68 (84.0)	25 (92.6)	0.35
NYHA functional class, n (%)				>0.99
I	0 (0)	0 (0)	0 (0)	
II	16 (14.8)	13 (15.8)	3 (11.1)	
III	91 (84.3)	68 (84.2)	23 (85.2)	
IV	1 (0.9)	0 (0)	1 (3.7)	
6-min walk test (n = 10)	259 ± 115	355 ± 160	217 ± 68	a
Coronary artery disease, n (%)	76 (70.4)	57 (70.4)	19 (70.4)	>0.99
Atrial fibrillation, n (%)	76 (70.4)	55 (67.9)	21 (77.8)	0.47
Medication, n (%)				
Beta-blocker	80 (74.1)	65 (80.3)	15 (55.6)	0.02^b^
ACE inhibitor/AT1 antagonist	50 (46.3)	43 (53.1)	7 (25.9)	0.02^b^
ARNi	32 (29.6)	26 (32.1)	6 (22.2)	0.47
MRA	69 (63.9)	53 (65.4)	16 (59.3)	0.65
SGLT-2 inhibitor	46 (42.6)	37 (45.7)	9 (33.3)	0.37
Diuretics	87 (80.6)	60 (74.0)	27 (100)	<0.01^b^
NOAC/OAC	82 (75.9)	61 (75.3)	21 (77.8)	>0.99
Tafamidis (n = 18)	13 (66.7)		18 (66.7)	
Amyloidosis types, n (%)				
ATTRwt			22 (81.5)	
ATTRv			1 (3.7)	
AL			4 (14.8)	
			27 (100)	
NAC staging, n (%)				
1			4 (17.4)	
2			10 (43.5)	
3			6 (26.1)	
4			3 (13)	
			23 (100)	
Mayo-Clinic staging, n (%)				
1			0 (0)	
2			1 (25)	
3a			2 (50)	
3b			1 (25)	
			4 (100)	
Device, n (%)				0.87
PASCAL	70 (64.8)	51 (62.9)	19 (70.4)	
PASCAL-10	6 (5.6)	5 (6.2)	1 (3.7)	
PASCAL-ACE	32 (29.6)	25 (30.9)	7 (25.9)	
Guide-sheet in to out (min)	80 (52-115)	83 (56-115)	64 (48-117)	0.38
Procedure time (min)	96 (62-128)	97 (65-138)	86 (52-118)	0.29
Fluoroscopy time (min)	12 (9-16)	12 (9-17)	11 (8-16)	0.29
Dose area product (cGycm^2^)	1,926 (1,190-3,085)	1,995 (1,202-3,054)	1,577 (1,046-3,176)	0.39

ACE = angiotensin-converting enzyme; AL = light chain amyloidosis; ARNi = angiotensin receptor-neprilysin inhibitor; ATTRv = variant transthyretin amyloidosis; ATTRwt = wild-type transthyretin amyloidosis; AT1 = angiotensin-1; BMI = body mass index; CA = cardiac amyloidosis; HF = heart failure; MRA = mineralocorticoid receptor antagonist; NAC = National Amyloidosis Centre; NOAC = Non-Vitamin K Antagonist Oral Anticoagulants; OAC = oral anticoagulants; SGLT-2 = sodium glucose linked transporter 2; TEER = transcatheter edge-to-edge repair.

a No statistical test due to small sample size (n = 10).

b Statistical significant at *P* < 0.05.

There were no differences in age (*P* = 0.81), sex (*P* = 0.35), body mass index (*P* = 0.59), EuroScore II (*P* = 0.69), and medical history for coronary artery disease (*P* > 0.99) or presence of atrial fibrillation (*P* = 0.47). Most patients were in NYHA functional class III or IV (CA 88.9% vs HF without CA 84.2%; *P* > 0.99). Median guide-sheet in-to-out time did not differ (CA group 64 [48-117] min vs HF group 83 [56-115] min; *P* = 0.38). Detailed baseline characteristics and procedure-related data are presented in Table 1.

SMD analysis of baseline characteristics showed good or excellent balance (SMD <0.2) for age (0.11), body mass index (0.06), EuroScore2 (0.06), presence of coronary artery disease (0.00), and estimated glomerular filtration rate (0.17). Potential imbalance was detected for gender (0.27), atrial fibrillation (0.22), left ventricular ejection fraction (0.30), SV (0.21), and N-terminal brain natriuretic peptide (0.32), with this difference most likely reflecting disease-specific characteristics of CA. Results are shown in Supplemental Table 1. Significant differences were observed between the 2 groups in specific laboratory values. N-terminal brain natriuretic peptide levels were markedly higher in the CA group, with a median of 4,419 (2,593-8,037) ng/L, compared to the HF group with 2,309 (853-5,307) ng/L (*P* = 0.003). High-sensitive cardiac troponin I levels were significantly elevated in the CA group, at 41 (27-69) ng/L, compared to 15 (7-33) ng/L in the HF group (*P* < 0.001). Other laboratory values showed no significant differences between the groups, including creatinine, estimated glomerular filtration rate, C-reactive protein, hemoglobin, and international normalized ratio (Table 2).

**Table 2:** Baseline Echocardiography and Laboratory Values

	All (N = 108)	HF + TEER (n = 81)	CA + TEER (n = 27)	*P* Value
Hemodynamics				
Blood pressure mean (mm Hg)	97.0 ± 15.0	98.8 ± 12.6	92.0 ± 12.2	<0.04^a^
Heart rate (beats/min)	70 (63-78)	70 (63-78)	73 (61-83)	0.72
Laboratory values				
hs-cTnI (ng/L)	23 (10-41)	15 (7-33)	40 (27-69)	<0.001^a^
NT-proBNP (ng/L)	2,910 (1,194-5,816)	2,309 (853-5,307)	4,419 (2,593-8,037)	<0.01^a^
Creatinine (mg/dL)	1.4 (1-1.6)	1.3 (1-1.6)	1.4 (1.1-1.7)	0.17
eGFR (mL/min/1.73 m^2^)	50.6 ± 19.7	51.3 ± 19.8	47.6 ± 19.4	0.38
CRP (mg/dL)	0.4 (0.4-1)	0.4 (0.4-1)	0.5 (0.4-1.3)	0.40
Hb (g/dL)	12.5 ± 1.9	12.4 ± 2.0	13.0 ± 1.8	0.14
INR	1.1 (1.0-1.3)	1.1 (1.0-1.3)	1.1 (1.1-1.3)	0.76
Echocardiography				
LVEF (%)	45.3 ± 13.0	46.4 ± 13.2	41.8 ± 11.7	0.11
Stroke volume (mL)	57 0.7 ± 22.2	63.0 ± 21.7	42.0 ± 15.7	<0.001^a^
LVEDD (cm)	5.5 ± 1.1	5.7 ± 1.1	5.0 ± 1.1	<0.01^a^
IVSd (cm)	1.4 ± 0.4	1.2 ± 0.3	1.8 ± 0.4	0.58
LAVI (mL/m^2^)	62.8 ± 34.7	66.1 ± 38.3	52.3 ± 17.0	0.10
Grade of mitral regurgitation, n (%)				0.99
MR 1+/2+	15 (13.9)	11 (13.6)	4 (14.8)	
MR 3+/4+	93 (86.1)	70 (86.4)	23 (85.2)	
Vena contracta (mm)	8.2 ± 2.4	8.4 ± 2.4	7.6 ± 1.4	0.19
Regurgitation volume (mL)	60.1 ± 24.3	60.7 ± 25.3	58.3 ± 21.3	0.71
EROA PISA (cm^2^)	0.4 ± 0.2	0.4 ± 0.2	0.5 ± 0.3	0.10
Mean transmitral gradient (mm Hg)	1.8 ± 1.0	2.0 ± 1.0	1.1 ± 0.2	<0.001^a^
TAPSE (cm)	2.1 ± 2.6	2.3 ± 3.0	1.5 ± 0.5	0.16
Grade of tricuspidal regurgitation, n (%)				>0.99
TR 1+/2+	76 (70.4)	56 (69.1)	20 (74.1)	
TR 3+/4+	32 (29.6)	25 (30.9)	7 (25.9)	
Pulmonary artery systolic pressure (mm Hg)	42.9 ± 13.8	44.4 ± 14.3	38.5 ± 11.4	0.08

CRP = C-reactive peptide; eGFR = estimated glomerular filtration rate; EROA = effective regurgitant orifice area; Hb = hemoglobin; hs-cTnI = high-sensitive cardiac troponin I; INR = international normalized ratio; IVsd = interventricular septal end-diastolic dimension; LAVI = left atrium volume index; LVEDD = left ventricular end-diastolic diameter; LVEF = left ventricular ejection fraction; MR = mitral regurgitation; NT-proBNP = N-terminal brain natriuretic peptide; PISA = proximal isovelocity surface area; TAPSE = tricuspid annular plane systolic excursion; TR = tricuspidal regurgitation; other abbreviations as in Table 1.

a Provocation by physical exercise (handgrip-echocardiography).

Most patients in both groups had a baseline MR grade of 3+/4+ without meaningful differences between the groups. In 13.9%, moderate MR was shown in TTE examination at rest which progressed to severe MR under physical stress by handgrip (dynamic MR 3+). The CA group had a notably lower left ventricular end-diastolic diameter, averaging 5.0 ± 1.1 cm, compared to 5.7 ± 1.1 cm in the HF group (*P* < 0.01). Similarly, SV was lower in the CA group, with an average of 42 ± 15.7 mL, compared to 63.0 ± 21.7 mL in the HF group (*P* < 0.001). MV mean pressure gradient was lower in the CA group than in the HF group (1.1 ± 0.2 vs 2.0 ± 1.0; *P* < 0.001). Other echocardiographic parameters including vena contracta, effective regurgitant orifice, left atrium volume index, and tricuspid annular plane systolic excursion did not show significant differences between the groups at baseline (Table 2).

There were no significant differences between anatomical complexity criteria in both groups according to the previously published criteria by Hausleiter et al (Table 3).^17^

**Table 3:** Anatomical Complexity Criteria

	All (N = 108)	HF + TEER (n = 81)	CA + TEER (n = 27)	*P* Value
≥2 independent Jets, n (%)	32 (29.6)	24 (29.6)	8 (29.6)	>0.99
Bileaflet/Multi Scallop prolapse involvement, n (%)	8 (7.4)	8 (9.9)	0 (0)	0.19
Significant Jet in the commissural area, n (%)	4 (3.7)	4 (4.9)	0 (0)	0.57
Flail width >15 mm and/or flail gap >10 mm, n (%)	0 (0)	0 (0)	0 (0)	
Other (Other includes presence of significant cleft or perforation in grasping area, moderate to severe calcification in the grasping), n (%)	19 (17.6)	13 (16.1)	6 (22.2)	0.56

Abbreviations as in Table 1.

### Outcome after TEER

**Figure 1:**
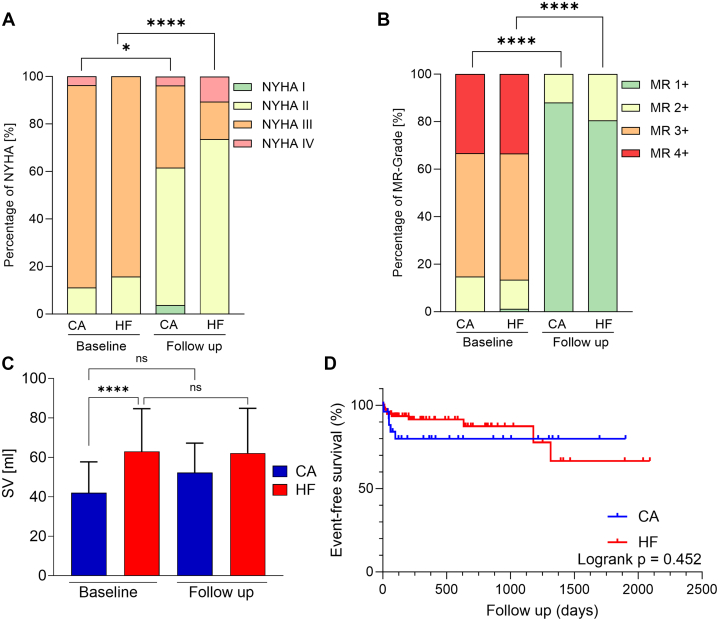
Overall Outcome in CA and HF Patients at Baseline and Follow-Up (A) NYHA functional class at baseline and at follow-up; (B) MR grade at baseline and at follow-up; (C) SV at baseline and at follow-up; (D) Overall survival probability following TEER as per last patient contact. ∗*P* < 0.05; ∗∗∗∗*P* < 0.0001. CA = cardiac amyloidosis; HF = heart failure; MR = mitral regurgitation; TEER = transcatheter edge-to-edge repair; SV = stroke volume.

Both groups were examined before intervention and after a median follow-up time of 96 (80-136) days (CA group) or 105 (93-147) days (HF group) after TEER (*P* = 0.22). The follow-up included both clinical evaluations and echocardiographic assessments to provide a comprehensive assessment of the patient's condition. An improvement in NYHA functional class was observed in the CA group (pre-TEER NYHA functional class ≥III: 24 [88.9%] patients vs post-TEER NYHA functional class ≥III: 10 [38.4%] patients; *P* < 0.01) and in the HF group (pre-TEER NYHA functional class ≥III: 65 [84.2%] patients vs post-TEER NYHA functional class ≥III: 19 [26.4%] patients; *P* = 0.001) (Figure 1A). There were no differences in laboratory values after TEER in CA group and HF group.

Postprocedural TTE assessment showed similar data in both groups. There was a reduction after intervention in MR grade (MR ≤2+) in the CA group (100%, *P* < 0.001) and HF group (100%, *P* < 0.001) without difference between the groups (*P* > 0.99; Figure 1B). Postprocedural mean pressure gradient showed an increase in both groups (*P* < 0.001), without exceeding a 6 mm Hg cutoff in any patient. There were no changes of SV after the intervention in any group (Figure 1C).

Long-term follow-up was achieved by the assessment of the last medical contact and status as per medical records. Within a median follow-up of 318 (109-777) days post-TEER, 2 CA group patients (cardiogenic shock and septic shock) and 4 HF group patients (septic shock, cardiogenic shock, subdural hematoma, and respiratory failure) died (*P* = 0.84) (Figure 1D).

**Figure 2:**
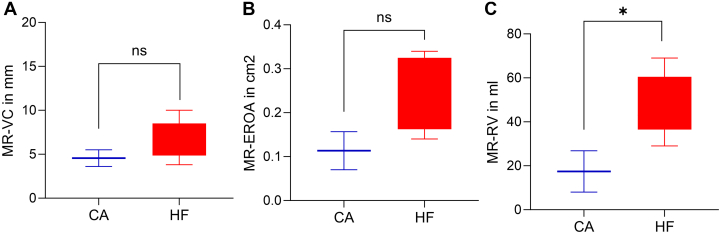
Postimplantation Comparison of Echocardiographic Parameters Between HF and Amyloidosis (A) Boxplots of MR-VC at follow-up; (B) Boxplots of MR-EROA at follow-up; (C) Boxplots of MR-RV at follow-up. ∗*P* < 0.05. MR-EROA = mitral regurgitant effective regurgitant orifice area; MR-VC = mitral regurgitant vena contracta; RV = regurgitant volume; other abbreviations as in Figure 1.

MR-effective regurgitant orifice area at follow-up did show a difference with a tendency toward numerically lower MR-effective regurgitant orifice area in the CA group as compared to the HF group (0.11 ± 0.1 cm^2^ vs 0.26 ± 0.1 cm^2^; *P* = 0.76). Vena contracta showed also no differences (CA 4.6 ± 1.3 mm vs HF 6.7 ± 2.2 mm; *P* = 0.65). RVol showed a difference with lower values in the CA group after TEER (CA 17.4 ± 13.3 mL vs HF 46.8 ± 14.4 mL; *P* = 0.05) (Figure 2).

All reported *P* values were derived from univariate statistical tests (eg, chi-square, Fisher exact, Mann-Whitney *U* test, or *t*-test where appropriate). No multivariable regression analyses were performed due to the limited sample size and the exploratory nature of the study.

The study demonstrated the safety and feasibility of TEER in amyloidosis patients, defined by the criteria from the MV academic research consortium.^24^ In both groups, we did not observe deaths, neurological events, myocardial infarction, or technical failure complications after intervention. In the CA group, 3 patients were hospitalized before the actual follow-up, 2 due to cardiac decompensation, and the other due to coronavirus disease 2019 pneumonia, while in the HF group, 5 patients required hospitalization prior to the scheduled follow-up due to cardiac decompensation. Six minor bleeding complications (drop in Hb <2 g/dL) were seen overall (Table 4).

**Table 4:** Safety Outcomes After TEER Intervention

	All (N = 108)	HF + TEER (n = 81)	CA + TEER (n = 27)	*P* Value
30-day mortality, n (%)	0 (0)	0 (0)	0 (0)	
Hospitalization before follow-up, n (%)	8 (7.4)	5 (6.1)	3 (11.1)	0.41
Decompensated HF, n (%)	7 (6.5)	5 (6.1)	2 (7.4)	
Noncardiovascular causes, n (%)	1 (0.9)	0 (0)	1 (3.7)	
Myocardial infarction, n (%)	0 (0)	0 (0)	0 (0)	
Stroke, n (%)	0 (0)	0 (0)	0 (0)	
Bleeding complications, n (%)	7 (6.5)	6 (7.4)	1 (3.7)	0.68
Minor (MVARC bleeding scale), n (%)	6 (5.6)	5 (6.2)	1 (3.7)	
Major (MVARC bleeding scale), n (%)	1 (0.9)	1 (1.2)	0 (0)	
Vascular complications, n (%)	0 (0)	0 (0)	0 (0)	
Arrhythmias, n (%)	0 (0)	0 (0)	0 (0)	
Infection at puncture side, n (%)	2 (1.8)	2 (2.4)	0 (0)	>0.99
Relevant iASD (Qp/Qs >1.4), n (%)	2 (1.8)	1 (1.2)	1 (3.7)	0.44

iASD = iatrogen atrium septum defect (Note: Qp/Qs > 1.4 = hemodynamically relevant); MVARC = mitral valve academic research consortium; other abbreviations as in Table 1.

## Discussion

This study examined the outcomes and safety profile of TEER for treating MR in patients with CA compared to those without. The main findings of this study are: 1) TEER offers a feasible alternative for high-risk CA patients with MR; 2) TEER in CA patients leads to symptom relief with improved NYHA functional class stage; 3) despite hypothesized complex cardiac anatomy in CA, procedural success was achieved in all cases; 4) TEER had a favorable safety profile in CA patients.

The importance of this investigation lies in the frequent comorbidity of MR and CA, which complicates diagnosis and treatment due to the diverse etiology and clinical manifestations associated with amyloid deposits in cardiac tissues. These deposits lead to structural abnormalities, such as valvular dysfunction, that significantly impair patient outcomes and exacerbate HF symptoms. The challenges of managing CA are underscored by the complexity of treating concomitant MR, which intensifies HF symptoms and complicates pharmacological treatment. The infiltration of amyloid proteins into the cardiac tissues not only disrupts normal cardiac function but also affects the heart's structural integrity, thus further increasing surgery-related risk. This study's findings highlight the potential of TEER as an alternative to surgical MV repair, especially for patients at high surgical risk, such as those with CA. These results are in line with earlier studies demonstrating the safety and feasibility of mitral TEER in patients with CA.^16^^,^^25^, ^26^, ^27^ Our study expands on these findings by including a larger patient cohort and a matched control group, allowing for a more robust comparison. Despite the higher baseline risk and advanced disease in CA patients, our data suggest that they can still experience meaningful clinical benefit from this procedure. TEER has emerged as a promising treatment modality due to its feasibility and safety in reducing MR severity and improving patients' functional status.

All patients included in the study were associated to a supraregional interdisciplinary amyloidosis center. Conscientious preprocedural screening for contraindications or difficulties to be anticipated is crucial to ensure the safety and effectiveness of the procedure, thereby helping to improve patient safety. It is likely that treatment of this specific patient population in high-capacity centers with profound experience and availability of all disciplines involved in the treatment of CA could improve procedural outcome. Future research should focus on larger patient cohorts and extended follow-up periods to better understand the potential benefits and refine patient selection criteria, aiming to optimize outcomes and ensure that TEER can be effectively integrated into the treatment protocols for patients with CA and MR.

### Study Limitations

The study has several limitations that should be considered when interpreting the findings. The retrospective nature of the analysis is a significant limiting factor. Although the number of patients with CA who received TEER in this study is the highest reported in the literature at 27 patients, more robust and generalizable results would require a larger sample size and prospective setting. The predominance of wild-type transthyretin amyloidosis patients in our cohort, with only a small number of AL amyloidosis cases, limits the generalizability of our findings to all CA subtypes. Differences in patients' characteristics between the groups including echocardiographic parameters at baseline may have introduced bias despite matching. Additionally, the long-term follow-up was based on the last medical contact and status as per medical records, which may underestimate potential adverse events due to the lack of standardized follow-up protocols. However, while NYHA functional class improvement was observed, we acknowledge that this subjective measure may be vulnerable to interobserver variability and potential bias. To address this limitation, future studies could incorporate more objective assessments of functional capacity, such as a HF-specific quality of life questionnaire, to complement NYHA functional class changes and provide a more comprehensive view of clinical improvement.

## Conclusions

This is the largest report on TEER for MR in CA patients. The feasibility of TEER in CA was demonstrated with a 100% procedural success rate and satisfactory outcome, noninferior to HF control patients. There was a favorable safety profile with no major procedure-related adverse events, thus providing a new therapeutic option in this vulnerable collective.

Perspectives **COMPETENCY IN PATIENT CARE AND PROCEDURAL SKILLS:** TEER is a feasible and safe alternative to surgical mitral valve repair in high-risk CA patients with severe MR, achieving high procedural success and symptom relief. Given the limited alternative treatment options, TEER should be considered a viable strategy in selected patients, potentially improving their functional capacity and quality of life. The management of CA patients undergoing TEER should be performed in high-volume specialized amyloidosis centers to enhance procedural safety and outcomes.

**TRANSLATIONAL OUTLOOK:** Future research should focus on larger, prospective studies with extended follow-up to refine patient selection criteria and optimize TEER outcomes in amyloidosis patients.

## Funding support and author disclosures

Dr Vogel has received personal fees outside the submitted work from Eli Lilly, Bayer, Alnylam, and Pfizer and has received research funding from UMEA Clinician Scientist Scholarship from the Faculty of Medicine, University of Duisburg-Essen, and 10.13039/100004326Bayer for an investigator/institution sponsored collaborative study outside the submitted work. Dr Luedike has received personal fees outside the submitted work from Edwards. Dr Mahabadi has received personal fees outside the submitted work from Amgen, Berlin Chemie, Daiichi Sankyo, Edwards Lifesciences Services, GmbH, Novartis, and Sanofi; has received research funding from 10.13039/501100022274Daiichi Sankyo; and is a cofounder of Mycor GmbH, a company focusing on artificial intelligence–based electrocardiography algorithms. Dr Settelmeier has received personal fees outside the submitted work from AstraZeneca; and has received research funding from UMEA Clinician Scientist Scholarship from the Faculty of Medicine, University of Duisburg-Essen. Dr Buehning has received research funding from UMEA Clinician Scientist Scholarship from the Faculty of Medicine, University of Duisburg-Essen. Dr Lerchner has received personal fees outside the submitted work from Bayer. Dr Mondritzki is a full employee of Bayer AG. Dr Carpinteiro has received personal fees outside the submitted work for consulting and/or lectures from Alexion, Alnylam, Amgen, GSK, Janssen, Pfizer, Sanofi, and Takeda and travel and congress participation grants from Janssen. Dr Reinhardt has received consulting and lecture fees outside the submitted work from AbbVie, AstraZeneca, Vertex, and Merck; has received research funding outside the submitted work from Gilead Pharmaceuticals and AstraZeneca; and is a cofounder of CDL Therapeutics GmbH. Dr Schindhelm has received personal fees outside the submitted work from Edwards Lifesciences Services, GmbH, MedMile, and Björn Steiger Stiftung. Dr Rassaf has received personal fees outside the submitted work from Novartis, Bristol Myers Squibb, Bayer, Daiichi Sankyo, AstraZeneca, and Pfizer; has received research funding from 10.13039/501100001659German Research Foundation (DFG; RA 969/12-1); and is a cofounder of Mycor GmbH, a company focusing on artificial intelligence–based electrocardiography algorithms. Dr Michel has received personal fees outside the submitted work from Bayer, Bristol Myers Squibb, Alnylam, AstraZeneca, IFFM e. V., and from Bund der Niedergelassenen Kardiologen (BNK); has received research funding from IFORES Clinician Scientist Scholarship from the Faculty of Medicine, University of Duisburg-Essen, and 10.13039/100004326Bayer for an investigator/institution sponsored collaborative study outside the submitted work. All other authors have reported that they have no relationships relevant to the contents of this paper to disclose.
