# Supplementary material for: Safety and Feasibility of Transcatheter Edge-to-Edge Repair of Mitral Regurgitation in Cardiac Amyloidosis
Source: JACC Adv. 2025 Jul 17;4(8):101998. doi: 10.1016/j.jacadv.2025.101998 (PMC12284670; doi:10.1016/j.jacadv.2025.101998)
Supplement: Supplementary data [file mmc1.docx]

***Supplemental Table 1: Standardized mean differences of baseline characteristics***

|  | **SMD** |
| --- | --- |
| **Variable** |  |
| Age | 0.11 |
| Male | 0.27 |
| BMI | 0.06 |
| EuroScore2 | 0.06 |
| Coronary artery disease | 0.00 |
| Atrial fibrillation | 0.22 |
| LVEF | 0.30 |
| SV | 0.21 |
| NT-proBNP | 0.32 |
| eGFR | 0.17 |

*BMI, body mass index; eGFR, estimated glomerular filtration rate; NT-proBNP, n-terminal brain natriuretic peptide; LVEF, left ventricular ejection fraction; SV, stroke volume.*
